# Transcriptional Program Induced by Wnt Protein in Human Fibroblasts Suggests Mechanisms for Cell Cooperativity in Defining Tissue Microenvironments

**DOI:** 10.1371/journal.pone.0000945

**Published:** 2007-09-26

**Authors:** Zach Klapholz-Brown, Graham G. Walmsley, Ysbrand M. Nusse, Roel Nusse, Patrick O. Brown

**Affiliations:** 1 Department of Biochemistry, Stanford University School of Medicine, Stanford, California, United States of America; 2 Department of Developmental Biology, Stanford University School of Medicine, Stanford, California, United States of America; 3 Howard Hughes Medical Institute, Stanford University School of Medicine, Stanford, California, United States of America; Baylor College of Medicine, United States of America

## Abstract

**Background:**

The Wnt signaling system plays key roles in development, regulation of stem cell self-renewal and differentiation, cell polarity, morphogenesis and cancer. Given the multifaceted roles of Wnt signaling in these processes, its transcriptional effects on the stromal cells that make up the scaffold and infrastructure of epithelial tissues are of great interest.

**Methods and Results:**

To begin to investigate these effects, we used DNA microarrays to identify transcriptional targets of the Wnt pathway in human lung fibroblasts. Cells were treated with active Wnt3a protein in culture, and RNA was harvested at 4 hours and 24 hours. Nuclear accumulation of ß-Catenin, as shown by immunofluorescence, and induction of *AXIN2* demonstrate that fibroblasts are programmed to respond to extracellular Wnt signals. In addition to several known Wnt targets, we found many new Wnt induced genes, including many transcripts encoding regulatory proteins. Transcription factors with important developmental roles, including *HOX* genes, dominated the early transcriptional response. Furthermore, we found differential expression of several genes that play direct roles in the Wnt signaling pathway, as well as genes involved in other cell signaling pathways including fibroblast growth factor (FGF) and bone morphogenetic protein (BMP) signaling**.** The gene most highly induced by Wnt3a was *GREMLIN2*, which encodes a secreted BMP antagonist.

**Conclusions:**

Elevated expression of *GREMLIN2* suggests a new role for Wnt signals in the maintenance of stem cell niches, whereby Wnt signals induce nearby fibroblasts to produce a BMP antagonist, inhibiting differentiation and promoting expansion of stem cells in their microenvironment. We suggest that Wnt-induced changes in the gene expression program of local stromal cells may play an important role in the establishment of specialized niches hospitable to the self-renewal of normal or malignant epithelial stem cells *in vivo*.

## Introduction

Wnt genes encode conserved, secreted signaling molecules that play important roles in numerous aspects of metazoan development, including stem cell regulation, fate determination, body axis specification, cell polarity, proliferation and differentiation [1]. The ‘canonical’ Wnt/ß-Catenin signaling pathway is initiated by the binding of Wnt proteins to Frizzled and LRP cell surface receptors, leading to the activation of an intracellular signal transduction pathway, initiated by Dishevelled (Dvl), which results in the inhibition of ß-Catenin phosphorylation by glycogen synthase kinase-3ß (GSK-3). ß-Catenin normally associates with a cytoplasmic scaffolding complex that includes Axin 2, Adenomatous polyposis coli (APC) and GSK-3, where it is phosphorylated by GSK-3 and subsequently targeted for ubiquitination and degradation in the proteasome complex [2]. However, in the presence of Wnt ligands, unphosphorylated ß-Catenin accumulates in the cytoplasm and enters the nucleus where it binds to TCF/LEF family of DNA binding proteins to activate Wnt target gene expression [2], [3]. Conversely, in the absence of Wnt ligands, competitive binding of the transcriptional co-repressor TLE1 (GROUCHO) to TCF represses transcription of Wnt target genes [2], [4].

The most abundant mesenchymal cells in the stroma of most tissues and organs are fibroblasts. These cells secrete extracellular matrix components and signaling molecules that contribute to the establishment of customized microenvironments for epithelial cells and providing a specialized niche for tissue stem cells. Mesenchymal-epithelial interactions are critical for the development of epithelial tissues and organs such as the skin, teeth, gut, and lungs [5]–[8], and play an important role in the inititation and progression of tumorigenesis [9].

In addition to the Wnt pathway, BMP signaling is also critical for the regulation of cell differentiation and suppression of abnormal proliferation. Inhibition of BMP signaling has been linked to abnormal developmental phenotypes in a number of organ systems. Transgenic expression of the BMP inhibitor Noggin has been shown to induce ectopic crypt formation in murine intestinal epithelia [10]. Moreover, conditional deletion of *BMPR1A,* which is normally expressed during branching morphogenesis in the mouse lung epithelium, induces defects in lung development, marked by increased apoptosis and morphological changes [11]. Coordination and spatial patterning of Wnt and BMP signaling plays an important role in the precise spatial and temporal regulation of differentiation and self-renewal of epithelial stem cells in the crypts and villi of the intestine [12].

During hair follicle development in the mouse, epithelial cells of the ectoderm secrete Wnt3a, leading to nuclear accumulation of ß-Catenin, and mesenchymal cells secrete Noggin, which activates the Lef1 transcription factor; Lef1 can subsequently form a heterodimer with ß-Catenin to regulate transcription of target genes [13]. Simultaneous Noggin and Wnt3a signals are required for expansion of the stem cell pool to promote downgrowth of the epithelial bud for proper hair follicle morphogenesis [13].

Recent work has shown that stromal fibroblasts in basal cell carcinomas and other diverse carcinomas express the secreted BMP antagonist *GREMLIN1,* at levels much higher than do stromal cells in the corresponding normal tissues [14]. Immunohistochemical analysis demonstrated that the basal cell carcinoma cells express BMP2 and BMP4, which are selectively inhibited by Gremlin. These data have led to the hypothesis that expression of secreted BMP antagonists by tumor-associated stromal cells may help shift the balance between proliferation and differentiation and promote self-renewal of cancer stem cells [14].

The gene expression program in the benign mesenchymal tumor desmoid type fibromatosis (DTF) hints at the possibility that Wnt signaling might play a role in the reciprocal interactions between the tumor stroma and neoplastic epithelium in carcinomas. Wnt-pathway activating point mutations of APC or ß-Catenin are commonly found in DTF soft tissue tumors, and a subset of breast carcinomas are characterized by a stromal gene expression signature that parallels the characteristic gene expression signature of DTF tumors [15]. The observation that this DTF signature is typically seen in a subset of human breast carcinomas that express high levels of *WNT2* mRNA [15], suggests that secreted Wnt signals from tumor cells may play a role in the distinct phenotype of tumor-associated fibroblasts and in the proliferation of fibroblasts accompanying the growth of carcinomas.

Dissecting the molecular mechanisms that underlie the cell to cell signaling and crosstalk between epithelial cells, stem cells, fibroblasts and other stromal cells in tissue microenvironments is of great importance in understanding important aspects of normal development and cancer progression. Here, we focus on one molecular program that may play a role in mesenchymal-epithelial interactions in development and cancer– the transcriptional response of fibroblasts to Wnt signals.

## Materials and Methods

### Cells and Reagents

CCL-186 (ATCC) primary fetal human lung fibroblasts (HLF) were removed from culture vessels with trypsin-EDTA and plated in RPMI medium with 5% fetal calf serum (FCS) supplemented with 1% glutamine. The cells were propagated at 37°C in 5% CO_2_ at a density of ∼4×10^6 ^cells per mL and grown to ∼90% confluence. Cells were split into six-well plates and grown to near confluence under the same conditions. Prior to the beginning of the timecourse, cells were switched to “low serum” medium (0.5% FCS) for ∼40 hours.

### Wnt3a Treatment Timecourse

In one of the two timecourse experiments, three wells received active Wnt3a protein (final concentration of 100 ng/ml Wnt3a) followed by incubation for 4 hours prior to harvesting, and three wells received active Wnt3a protein followed by incubation for 24 hours before harvesting. Three wells were treated with the same volume of the vehicle solution, and harvested at 4 hours as “mock 4” negative controls and three wells were treated with an equal volume of vehicle and harvested at 24 hours as “mock 24” samples. Additionally, six samples received no treatment and were harvested at the beginning of the timecourse as “time zero” samples. In a second independent replicate experiment under the same culture conditions, samples included one “time zero”, one “mock 4”, two “mock 24”, one “Wnt treatment 4 hour” and two “Wnt treatment 24 hour” samples. Data from each experiment were combined in subsequent analyses.

### RNA Isolation and cDNA synthesis

For each of the two growth conditions (Wnt3a^+^ medium, Wnt3a^− ^medium), cells were lysed by addition of TRIZOL reagent (Invitrogen, Carlsbad, CA) and total RNA isolated according to the manufacturer's protocol. Poly-adenylated RNAs from each sample, as well as a reference mRNA pool (Stratagene), were amplified and labeled with amino-allyl-dUTP, using the Amino Allyl MessageAmp II aRNA kit (Ambion Cat# 1753), following the manufacturer's instructions. The amino-allyl-dUTP-labeled amplified RNA samples were then fluorescently labeled by coupling to Cy5-NHS (for the experimental samples) or Cy3-NHS (for the reference RNA). The differentially labeled experimental and reference RNAs were mixed together and analyzed by comparative hybridization to HEEBO DNA microarrays (http://microarray.org/sfgf/heebo.do) as described (http://cmgm.stanford.edu/pbrown/protocols/index.html). The HEEBO microarrays contain 44,544 70-mer oligonucleotide probes, representing approximately 30,718 unique genes. A detailed description of these arrays can be found at (http://microarray.org/sfgf/heebo.do) and the identity and origin of each probe on these arrays is detailed in Supplemental Table S1. Arrays were scanned using the Genepix4000B scanner (Axon Instruments, Molecular Devices Corp. Palo Alto, CA), and data were deposited into the Stanford Microarray Database (SMD). The web supplement can be found at the SMD website (http://genome-www5.stanford.edu/).

### Data Analysis

From each array, data were selected for further analysis using SpotReader (Niles Scientific, San Francisco, CA), excluding data from array elements (corresponding to gene-specific probes) with obvious defects based on automated and visual inspection of the scanned arrays, and requiring that the fluorescence signal in either the Cy3 or Cy5 channel be at least twice the median of the local background. Data from the two replicate timecourse experiments were combined. Only genes for which the microarray data passed our data quality and signal/background criteria for at least 2/3 of the samples were considered for further analysis. We used Significance Analysis of Microarrays (SAM) software [16] to select for differentially expressed genes in HLF following Wnt-treatment at 4 hours, 24 hours, or both, in Wnt-treated cells, compared to the corresponding mock-treated cells and time-zero (untreated) cells. A false-discovery rate (FDR) of less than 1% was used as the cut-off for gene selection.

### ß-Catenin immunofluorescence

Human dermal fibroblasts were plated in six wells of a multiwell slide for antibody staining; four of the wells received Wnt3a treatment for 4 hours, and the remaining two received the diluent solution as negative controls. Cells were fixed in 4% paraformaldehyde and stained with anti-ß-Catenin primary, and fluorescein-labeled secondary antibodies. Cell nuclei were counterstained with DAPI.

## Results and Discussion

Previous studies of the transcriptional response of embryonic carcinoma cells have led to the identification of novel transcriptional targets of Wnt3a, many of which are known to play critical roles in embryonic stem cell control and embryogenesis [17]. A recent study identified transcriptional targets of Wnt/ß-Catenin signaling in mouse L929 fibroblasts after inducible expression of ß-Catenin or Dvl [18]. A comprehensive list of previously reported Wnt target genes can be found on the Wnt homepage: http://www.stanford.edu/∼rnusse/pathways/targets.html. Gene expression datasets of Wnt responses in other cell types can also be found at the Stanford Microarray Database website: http://genome-www5.stanford.edu/.

**Figure 1:**
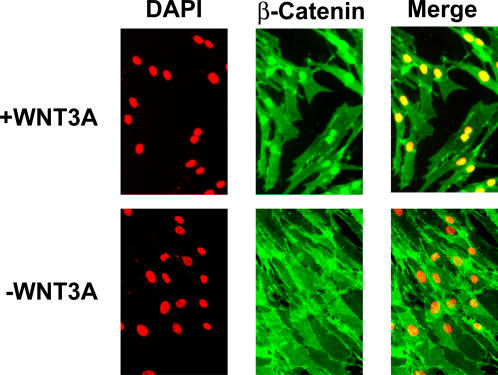
Immunofluorescence analysis comparing ß-Catenin localization in Wnt-treated and mock-treated human dermal fibroblasts. Immunofluorescence staining shows accumulation of nuclear ß-Catenin in Wnt-treated cells, but no expression in “mock” treated cells (after 4 hrs of Wnt treatment). DAPI was used to stain cell nuclei (red).

**Figure 2:**
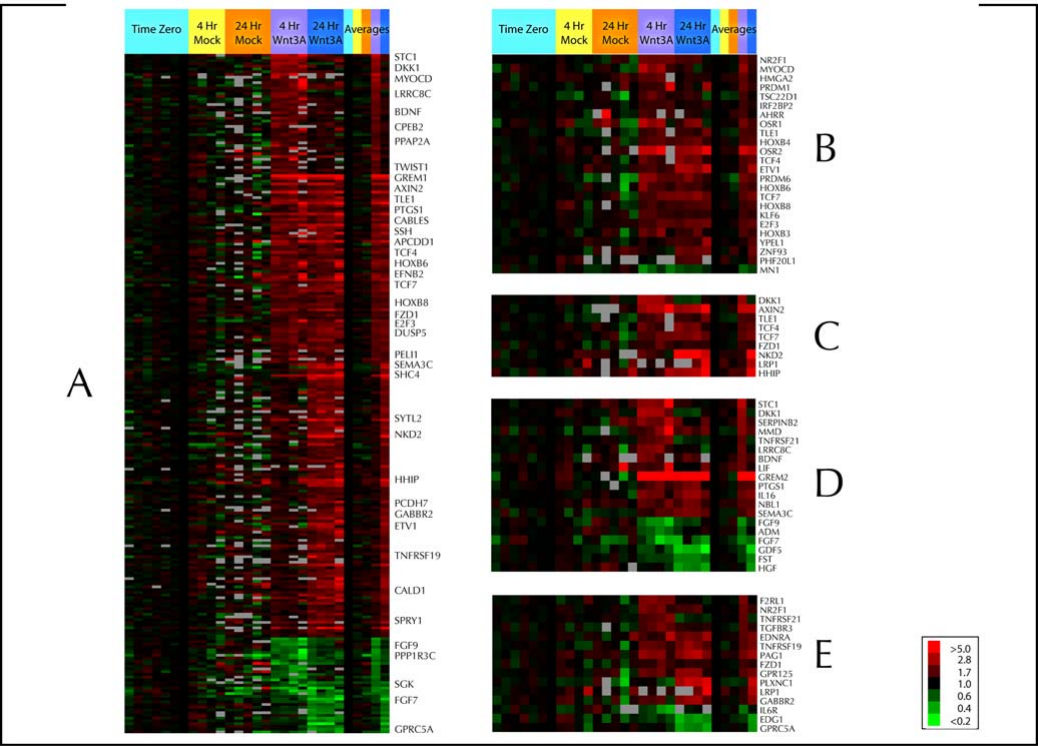
Wnt-regulated genes in human lung fibroblasts. A) SAM analysis was performed to select for genes that were differentially expressed in at 4 hour, 24 hour or both (averages), in Wnt-treated cells compared to the corresponding time zero and mock-treated fibroblasts, using an FDR of less than 1% as the cut-off. In this display, each row represents a specific gene, and each column a sample from the Wnt-treatment time course experiment. A red color indicates an increase in transcript levels of a given gene relative to the pre-treatment (time zero) level and green indicates decreased transcript levels relative to time zero. Grey represents missing or excluded data. The legend bar maps color versus magnitude of changes in transcript levels relative to the pre-treatment level. A version of this figure with every row labeled by gene name is available as Supplemental Figure S1. B) Shows expression patterns of Wnt-responsive genes encoding DNA binding proteins/transcription factors and transcriptional cofactors. C) Shows expression patterns of Wnt-responsive genes encoding proteins with direct roles in the Wnt signaling system. D) Shows expression patterns of Wnt-responsive genes encoding extracellular signaling molecules. E) Shows expression of Wnt-responsive genes encoding cell surface molecules and receptors.

We wanted to examine the transcriptional response of human fibroblasts to Wnt3a stimulation using DNA microarrays. To test whether fibroblasts are able to respond to Wnt, we used immunofluorescence analysis for nuclear ß-Catenin localization. Treatment of human dermal fibroblasts with Wnt3a for 4 hours at a concentration of 100ng/ml led to nuclear accumulation of ß-Catenin (Figure 1). Wnt3a treatment of HLFs in culture resulted in induction or repression of several genes that have been identified as Wnt targets in other cell types (e.g. *AXIN2, DKK1, TWIST1, MMP11*), as well as many genes not previously identified as targets of Wnt signal transduction (Figure 2A). We identified 215 genes that were consistently induced or repressed in response to Wnt3a, after either 4 or 24 hours, or both, using significance analysis of microarrays (SAM), with a false-discovery rate (FDR) of less than 1% (Supplemental Dataset S1, Supplemental Figure S1). Comparison of the responses at 4 and 24 hours provides a rough means of distinguishing genes that are more likely to be direct targets from those that are more likely to be secondary, downstream targets of Wnt signal transduction (Figure 2A).

### Many Transcription factors are regulated in response to Wnt

A notable feature of the early transcriptional response of HLF (Figure 2A) to Wnt signals was the induction of multiple DNA-binding proteins, including transcription factors as well as transcriptional cofactors (Figure 2B). Many of these are known to play important roles during cancer progression or in developmentally important processes such as differentiation and morphogenesis (e.g. *TWIST, NR2F1, MYOCD, PRDM1, PRDM6, KLF6, HMGA2*), developmental patterning (e.g. *HOXB3, HOXB4, HOXB6, HOXB8, OSR1, OSR2)*, regulation of cell-cell signaling (e.g. *NR2F1, TCF4, TCF7, AHRR, IRF2BP2, TSC22D1, TLE1)* and cell proliferation (e.g. *E2F3, ETV1*). The prominence of transcription factors and other regulatory genes in the transcriptional response at 4 hours suggests that a more extensive reprogramming of transcription would occur downstream of primary target gene activation; the mechanism of target gene activation is likely to be far more complex than direct activation through TCF/ß-catenin, involving a cascade of downstream transcription factors and regulatory proteins that play a role in further regulation of secondary Wnt target genes.

A prominent feature of the transcriptional response was the induction of genes with direct roles in the regulation of Wnt signaling (Figure 2C), either as inhibitors (e.g. *AXIN2, TLE1, LRP1, NKD2, DKK1, HHIP*) or as positive regulators (e.g. *TCF4, TCF7, FZD1*). Co-expression of both negative and positive regulators of Wnt signaling provides a network of opposing control mechanisms that are likely to contribute to the robust and precise regulation of Wnt target gene expression.

### Wnt induced expression of specific Hox genes

Among the transcription factors regulated by Wnt signal transduction, it was interesting to note that several *Hox* genes, which play important roles in pattern formation, morphogenesis and body-axis specification, featured prominently in the early transcriptional response (Figure 2B). It has recently been established that fibroblasts from different anatomic sites are characterized by a distinct positional identity, marked by unique embryonic “*Hox* codes” that may be retained from embryogenesis through adulthood [19]. Interestingly, both *HOXB3* and *HOXB6* mRNAs have previously been shown to be highly expressed in fetal lung fibroblasts [19]. *HOXB4* is induced by the canonical Wnt pathway in hematopoietic stem cells and plays an important role in promoting their expansion and self-renewal [20]. During lung development in the chick embryo, *HoxB* transcription factors show unique patterns of expression in the mesenchyme of distinct differentiated lung compartments [21]. Consistent with the potential role of Hox genes in the maintenance of a positional memory of fibroblasts into adulthood, Wnt signaling may enhance the transcriptional effects of the Hox gene code at critical stages of development and in adult tissue homeostasis.

### Connectivity between Wnt signaling and other cell signaling pathways

Many genes that play roles in other cell-signaling pathways, including the BMP and TGF-ß pathways (e.g. *GDF5, FST, TGFBR3, TGFB2*, *GREM2*), Hedgehog signaling (e.g. *HHIP*), MAPK signaling (e.g. *DUSP5, SSH2*) and FGF signaling (e.g. *SPRY1, FGF7, FGF9*), as well as genes encoding other extracellular signals (e.g. *LIF, HGF, SEMA3C, BDNF, STC1, IL16*) and cell-surface molecules or receptors (e.g. *IL6R, EDG1, PLXNC1, EDNRA, TNFRSF19*) were also regulated in response to Wnt signals in HLF (Figure 2D, Figure 2E). Local regulation by Wnt signals of diverse cell signaling pathways in fibroblasts could therefore have multifaceted consequences for tissue microenvironments *in vivo,* including the balance between differentiation and self-renewal, cell migration and adhesion. The interconnectivity of these regulatory systems may contribute to the robustness of stem cell niches and the precise spatial and temporal control of differentiation *in vivo*.

### *GREMLIN2* mRNA is strongly induced by Wnt3a in HLF

**Figure 3:**
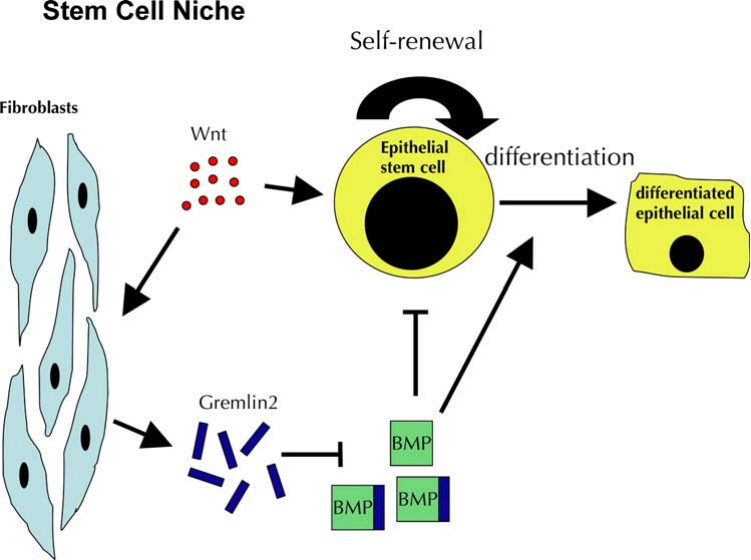
A possible dual role for Wnt in the maintenance of stem cell niches. We hypothesize that Wnt may promote self-renewal directly by raising nuclear ß-Catenin levels in stem cells and indirectly by activating the BMP inhibitor Gremlin 2 in nearby fibroblasts, thereby blocking target BMPs from promoting differentiation and loss of self-renewal capacity.

Interestingly, the gene most highly induced by Wnt3a was *GREMLIN2*, which encodes a secreted bone morphogenetic protein (BMP) antagonist. (Figure 2E). In a related experiment, PRDC, the mouse homolog of *GREMLIN2,* was identified as a transcriptional target of Wnt/ß-Catenin signaling after inducible expression of ß-Catenin or DVL in L929 mouse fibroblasts [18]. We suggest a model in which secretion of selective BMP antagonists by fibroblasts in response to local Wnt signals *in vivo* has an important role in inhibiting differentiation and promoting self-renewal of stem cells in some stem-cell niches, and perhaps in the tumor microenvironment (Figure 3). Secreted Wnt signals may serve a dual function in the regulation of diverse cellular niches by increasing ß-Catenin levels in target stem or progenitor cell populations to promote self-renewal, and coordinately activating *GREMLIN2* or other BMP antagonists in nearby stromal cells to block differentiation of stem cells in their microenvironment.

Although Wnts are known to play an important role in the maintenance of an undifferentiated state and the control of stem cell self-renewal, activation of ß-Catenin alone is not sufficient to promote self-renewal of mouse embryonic stem (mES) cells [22]. Recent data suggests that Wnt3a can act synergistically with leukemia-inhibitory factor (LIF) to maintain an undifferentiated state and self-renewal of mES cells [22]. LIF has been shown to activate BMP2 in neural stem cells, which leads to activation STAT3 and SMAD1 transcription factors to promote differentiation into astrocytes [23]. These data suggest that combinatorial signaling of LIF with either Wnts or BMPs can either block or promote differentiation, respectively. Interestingly, *LIF* was among the many genes encoding extracellular signaling molecules (Figure 2D) induced by Wnt3a in HLF, which further suggests that coordination between Wnt signals and other secreted signals contributes to the regulation of stem cell self-renewal in tissue microenvironments. Co-expression of *GREMLIN2* and *LIF* mRNA in lung fibroblasts suggests the possibility of a combinatorial mechanism for maintenance of self-renewal, whereby Gremlin 2 inhibits BMP2 in some stem cell niches, to favor the cooperativity between Wnt and LIF at the expense of BMP2/LIF mediated differentiation.

### Cell type specific regulation of Follistatin expression

Interestingly, the Follistatin (*FST*) gene, which encodes a secreted inhibitor of BMP and TGF-beta signaling, is a direct target of Wnt signal transduction in embryonic carcinoma cells, in which its induction by Wnt is dependent on a TCF binding site [17]. In HLF, however, we found that FST expression was markedly decreased in response to Wnt3a, which underscores the potential importance of cell-type specific variation in the Wnt response. This result, along with the observed induction of *TLE1* (GROUCHO) by Wnt3a, raises the possibility that the *FST* promoter may have cell-type specific TCF binding sites that specifically interact with TCF-ß-Catenin heterodimers to activate *FST* expression in embryonic carcinoma cells and with TCF-Groucho heterodimers to repress *FST* expression in HLF.

### Suppression of FGF signaling by Wnt and its implications for lung development

Both *FGF7* and *FGF9* were repressed in HLFs in response to Wnt3a treatment, whereas *Sprouty1* (*SPRY1*), which encodes an inhibitor of FGF signaling, was induced. This suggests that in these cells, the FGF signaling pathway is inhibited by Wnt signal transduction (Figure 2D). Conversely, several FGFs, including *FGF9*, are up-regulated by Wnt in other cell types, highlighting the diversity of cell-type specific responses to Wnt [24]–[26].

In the developing rat lung, mesenchymal FGF7 expression is required for the induction of branching morphogenesis of distal lung endoderm and the maintenance of an alveolar epithelial cell phenotype [5]. In the absence of mesenchymal FGF7 signals, alveolar differentiation is likely to be repressed, maintaining an undifferentiated state of distal lung endoderm. Thus, repression of FGF signaling by local Wnt signals may be an additional mechanism for local control of differentiation-during lung development, a Wnt signaling center in the proximal lung epithelium may inhibit alveolar differentiation by inhibition of mesenchymal FGF signaling, to maintain a bronchial epithelial stem cell niche.

### Wnt signals may contribute to a myofibroblast phenotype in fibroblasts

Among the genes differentially expressed in response to Wnt stimulation, genes that play a role in differentiation of fibroblasts to a myofibroblast or smooth muscle phenotype (e.g. *MYOCD*), or in promoting epithelial-mesenchymal transitions (e.g. *TWIST1*), were apparent (Figure 2). The transcription factor Myocardin (*MYOCD*), which plays a key role in differentiation of smooth muscle cells and has been implicated in myofibroblast differentiation [27], may activate a myofibroblast differentiation program in fibroblasts. Indeed, mRNA encoding Caldesmon (*CALD1)*, a marker of smooth muscle and myofibroblast differentiation, was induced at 24 hours after Wnt3a treatment. Co-expression of several genes with important roles in extracellular matrix degradation or cell adhesion (e.g. *MMP11, SEMA3C, EPHB2*) along with the transcription factors *MYOCD* and *TWIST1* and markers of smooth muscle or myofibroblast differentiation (e.g. *CALD1*) in response to Wnt signals, may indicate an important role for secreted Wnt signals in the regulation of mesenchymal cell differentiation and migration in processes such as wound healing and cancer progression.

### Concluding remarks

Throughout embryonic development and into adulthood, cells are exposed to diverse signals from their microenvironment that influence their proliferation, migration and developmental fate. The complex interplay among these molecular signals and the responses they elicit from the diverse cells that comprise tissue microenvironments is an important frontier in our understanding of basic mechanisms of development, adult tissue homeostasis, and cancer. Understanding the biological setting of the Wnt-response program of fibroblasts and the precise role that Wnt-regulated genes in these cells play in cell-cell signaling during embryonic development, normal physiology, and tumor progression *in vivo* will play an important part in advancing that frontier. This study contributes to that understanding by defining a transcriptional program in human lung fibroblasts.

Many questions raised by our results remain to be investigated. Exploration of the mechanisms of target gene regulation, by analysis of transcription factor binding sites, ectopic expression and RNAi knock-down of Wnt targets, should expand our mechanistic understanding of the transcriptional response of fibroblasts to Wnt signals. Preliminary data, examining the response of human dermal fibroblasts to Wnt3a, suggests that the responses of fibroblasts in different microenvironments to Wnt signals may include both shared features and important differences (Supplemental dataset S2 and Supplemental Figure S2 and Supplemental dataset S3 and Supplemental Figure S3). Observed differences in gene expression between the two fibroblasts types in response to Wnt are likely to due to the fact that fibroblasts from different anatomic sites are distinct differentiated cell types with programmed epigenetic differences and characteristic site-specific gene expression patterns [19]. Cell-type specific differences in responses to Wnt signals, including variations in the Wnt responses of fibroblasts from different tissues and organs, or fibroblasts at different stages of development (e.g. fetal vs. adult) could have an important influence on their ability to establish microenvironments hospitable to the growth of normal or cancer stem cells, and thus could have significant implications both in organogenesis, tissue homeoastasis and cancer pathogenesis. The diversity of stromal cell responses to Wnt is thus an important question for further investigation. Some Wnt signals may act as developmental switches that lead to long-term or permanent transcriptional reprogramming in target cells. Thus, another intriguing question that remains to be answered is whether and to what extent the Wnt-induced gene expression program in fibroblasts depends on continuing exposure to Wnt signals, or represent epigenetic changes (e.g. changes in DNA methylation or histone modifications) that lead to permanent transcriptional reprogramming.

## Supporting Information

Figure S1 Tiff image file of a treeview display of supplemental dataset S1.
(4.50 MB TIF)

Figure S2 Tiff image file of a treeview display of supplemental dataset S2.
(9.10 MB TIF)

Figure S3 Tiff image file of a treeview display of supplemental dataset S3.
(11.15 MB TIF)

Table S1 Each oligonucleotide on the microarrays used in this study is represented by a row of data in this table. The oligonucleotides are listed by sequence ID. For each oligonucleotide, the nucleotide sequence, LocusLink ID, genome location, gene name and symbol (and synonyms) are provided in the indicated columns (where applicable).
(15.62 MB XLS)

Dataset S1 Data from Figure 2A in Treeview (.pcl) file format. This file can be opened as a spreadsheet or using Treeview (http://rana.lbl.gov/EisenSoftware.htm), Java Treeview [28], or similar programs that allow visual display and browsing of the data. For each gene, in each cell sample, transcript levels are represented as the Log2 (expression level of that gene in the particular cell sample/average expression level of that gene in the time zero samples). In other words, they represent the level of expression in any sample relative to their expression level at the start of the Wnt- (or mock-) treatment time course.
(0.05 MB XLS)

Dataset S2 Selected data from time courses of Wnt3A treatment of human abdominal dermal fibroblasts. Note that the technical quality of the data from these experiments was significantly lower than the quality of the HDF experiment data and should be regarded as preliminary and inconclusive. The data are in Treeview (.pcl) file format. This file can be opened as a spreadsheet or using Treeview (http://rana.lbl.gov/EisenSoftware.htm), Java Treeview [28], or similar programs that allow visual display and browsing of the data. This dataset combines data from 3 independent timecourse experiments to characterize the transcriptional response of primary human abdominal dermal fibroblasts to Wnt3a treatment. In the first experiment, we only characterized the transcriptional response after 4 hours of treatment with Wnt3a using cDNA microarrays from the Stanford Functional Genomics Facility. In the second experiment we looked at the response after 24 hours of Wnt3a treatment with SH cDNA microarrays. In the third experiment we used HEEBO oligonucleotide microarrays (the same design used for the HLF experiments) to characterize the response after both 4 and 24 hours of Wnt3a treatment in parallel. The first experiment consisted of 3 “time zero” samples, two “mock-treated” controls and four Wnt-treated samples (all harvested at 4 hours). The second experiment consisted of two “time zero” samples, two “mock treated” control samples and three Wnt-treated samples (all harvested at 24 hours). The third experiment consisted of three “time zero” samples, two “mock-treated” controls, two Wnt-treated (4 hr) samples and two Wnt-treated (24 hr) samples. In all experiments, cells were propagated in DMEM medium with 10% fetal bovine serum (FBS), supplemented with 1% glutamine at 37 degrees C in 5% CO2 at a density of ∼4×106 cells per mL and grown to ∼90% confluence before treatment. ∼40 hours before initiation of the Wnt-treatment timecourse (exact times may have varied in different experiments), cells were switched to “low serum medium” (DMEM with 0.5% FBS). Cells were treated with Wnt3a protein at a final concentration of 100 ng/ml in all 3 experiments. To select genes differentially expressed in response to Wnt, the expression values for each gene (after excluding data with technically inadequate measurements) were transformed to base 2 log of the ratio of their expression levels to the mean expression level in the time zero samples from the corresponding experiment. The data from all three experiments were then combined for analysis and display. SAM was used to select genes differentially expressed in the Wnt-treated HDF cells at either 4 hours, 24 hours or both, relative to both the time zero samples and the corresponding mock-treated controls, at a false discovery rate (FDR) less than 5%.
(0.03 MB ZIP)

Dataset S3 Data from the Wnt3A treatment time courses for human lung fibroblasts (HLF) and human dermal fibroblasts (HDF) were combined, and SAM was used to select genes differentially expressed in the Wnt-treated cells relative to both the time zero samples and the corresponding mock-treated cells, at either 4 hours, 24 hours or both, in the combined datasets (i.e. combining all the time zero, 4 hour and 24 hour samples, respectively, from both cell types), at a false-discovery rate of less than 5%. The data are in Treeview (.pcl) file format. This file can be opened as a spreadsheet or using Treeview (http://rana.lbl.gov/EisenSoftware.htm), Java Treeview [28], or similar programs that allow visual display and browsing of the data.
(0.08 MB ZIP)
