# Supplementary figures and images for: Transcriptional Program Induced by Wnt Protein in Human Fibroblasts Suggests Mechanisms for Cell Cooperativity in Defining Tissue Microenvironments
Source: PLoS One. 2007 Sep 26;2(9):e945. doi: 10.1371/journal.pone.0000945 (PMC1976560; doi:10.1371/journal.pone.0000945)

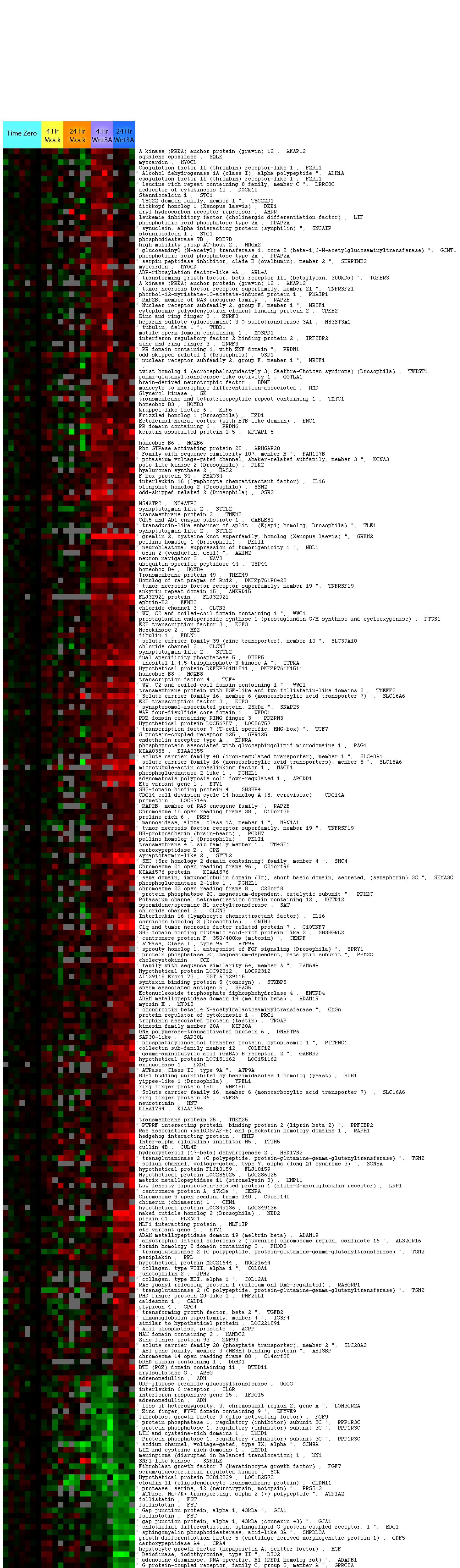

Supplement: Figure S1 — Tiff image file of a treeview display of supplemental dataset S1. (4.50 MB TIF) [file pone.0000945.s001.tif]

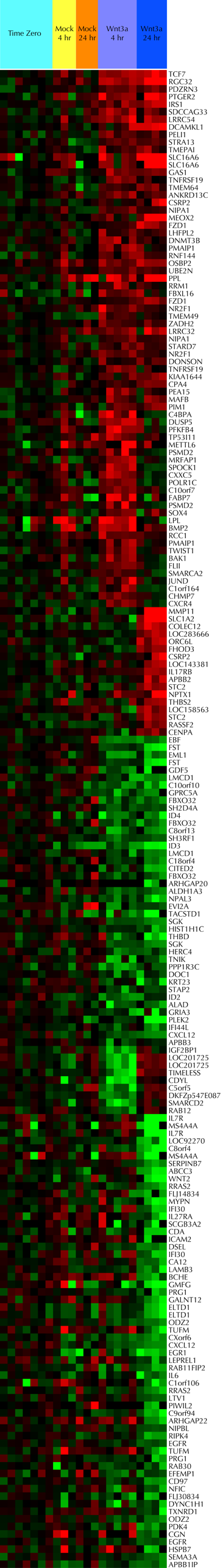

Supplement: Figure S2 — Tiff image file of a treeview display of supplemental dataset S2. (9.10 MB TIF) [file pone.0000945.s002.tif]

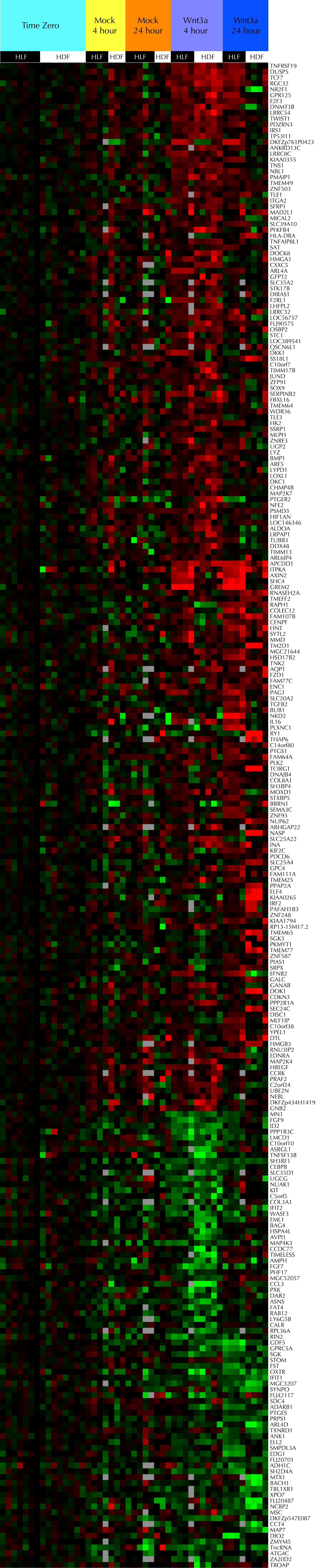

Supplement: Figure S3 — Tiff image file of a treeview display of supplemental dataset S3. (11.15 MB TIF) [file pone.0000945.s003.tif]
